# Supplementary material for: Burden and Trends of Diet-Related Colorectal Cancer in OECD Countries: Systematic Analysis Based on Global Burden of Disease Study 1990–2021 with Projections to 2050
Source: Nutrients. 2025 Apr 10;17(8):1320. doi: 10.3390/nu17081320 (PMC12029645; doi:10.3390/nu17081320)
Supplement: Supplementary file 1 [file nutrients-17-01320-s001.zip › nutrients-3568204-supplementary.pdf]

Supplementary Table S1: **Dietary risk factor exposure definitions and optimal level of exposure as defined by GBD 2021**

Dietary risk factor	Definition of exposure	Optimal level or range of intake
Diet low in whole grains	Average daily consumption (in grams per day) of whole grains (bran, germ, and endosperm in their natural proportion) from breakfast cereals, bread, rice, pasta, biscuits, muffins, tortillas, pancakes, and other sources	160–210 g/day
Diet low in milk	Average daily consumption (in grams per day) of dairy milk including non-fat, low-fat, and full-fat milk, but excluding plant-based milks, fermented milk products such as buttermilk, and other dairy products such as cheese	280–340 g/day (males)<br>500–610 g/day (females)
Diet high in red meat	Average daily consumption (in grams per day) of unprocessed red meat including pork and bovine meats such as beef, pork, lamb,<br>and goat, but excluding all processed meats, poultry, fish, and eggs	0–200 g/day
Diet high in processed meat	Average daily consumption (in grams per day) of meat preserved by smoking, curing, salting, or addition of chemical preservatives	0 g/day
Diet low in fibre	Average daily consumption (in grams per day) of fibre from all sources including fruits, vegetables, grains, legumes, and pulses	22–25 g/day
Diet low in calcium	Average daily consumption (in grams per day) of calcium from all sources, including milk, yoghurt, and cheese	0.72–0.86 g/day (males)<br>1.1–1.2 g/day (females)

Supplementary Table S2: Diet-related number, age-standardised percentage, and rate (per 100,000) of diet-related CRC death in 2021 among OECD countries

Country	Number (95% UI)			Age-standardised percentage (95% UI)			Rank *	ASDR (95% UI)		
	Male	Female	Both	Male	Female	Both		Male	Female	Both
OECD	79252 (16753, 126744)	75291 (21711, 116584)	154543 (37765, 243081)	37.6 (7.9, 59.8)	40.8 (11.5, 62.5)	39.1 (9.5, 61.0)		7 (1, 11)	5 (1, 7)	6 (1, 9)
Australia	1377 (274, 2251)	1261 (333, 2039)	2638 (606, 4237)	38.1 (7.6, 60.7)	41.3 (10.6, 63.7)	39.5 (9, 62.3)	11	6 (1, 10)	5 (1, 8)	6 (1, 9)
Austria	479 (92, 775)	424 (102, 679)	903 (192, 1451)	37.2 (7.3, 59.7)	40.1 (9.9, 62.3)	38.5 (8.4, 60.9)	17	6 (1, 9)	4 (1, 6)	5 (1, 7)
Belgium	739 (136, 1197)	720 (169, 1157)	1459 (301, 2332)	38.5 (7.2, 61.4)	41.2 (9.9, 63.7)	39.7 (8.4, 62.5)	9	7 (1, 11)	5 (1, 7)	6 (1, 9)
Canada	2111 (443, 3443)	2048 (547, 3225)	4159 (989, 6645)	37.3 (8.0, 59.7)	40.2 (11.6, 62)	38.7 (9.6, 60.9)	15	6 (1, 10)	5 (1, 7)	5 (1, 9)
Chile	701 (178, 1110)	775 (250, 1166)	1476 (428, 2274)	41.5(11.0, 64.2)	44.9 (15.0, 66.4)	43.2 (13.0, 65.3)	1	6 (2, 10)	5 (2, 8)	6 (2, 9)
Colombia	933 (274, 1538)	1210 (432, 1876)	2142 (720, 3374)	34.9 (11.1, 54.4)	38.8 (15, 57.9)	37.0 (13.2, 56.3)	24	4 (1, 6)	4 (1, 6)	4 (1, 6)
Costa Rica	142 (40, 235)	157 (55, 244)	299 (95, 473)	33.8 (10.0, 53.5)	37.9 (13.8, 56.9)	35.9 (11.8 , 55.4)	26	6 (2, 9)	5 (2, 8)	5 (2, 9)
Czechia	946 (209, 1585)	717 (200, 1126)	1663 (417, 2699)	35.4 (7.6, 57.4)	38.7 (10.8, 60)	36.8 (8.9, 58.5)	25	10 (2, 17)	5 (1, 9)	7 (2, 12)
Denmark	485 (88, 789)	485 (110, 772)	969 (194, 1558)	38.3 (7, 61.4)	41 (9.6, 63.6)	39.6 (8.2, 62.5)	10	9 (2, 14)	7 (2, 11)	8 (1, 12)
Estonia	104 (14, 173)	109 (20, 175)	213 (34, 348)	39.1 (5.7, 62.9)	41.6 (8.1, 64.9)	40.4 (6.8, 63.9)	4	10 (1, 17)	5 (1, 9)	7 (1, 12)
Finland	302 (58, 495)	306 (71, 492)	608 (126, 991)	37.3 (7.2, 60.2)	39.8 (9.6, 61.7)	38.4 (8.2, 61.0)	18	5 (1, 8)	4 (1, 6)	4 (1, 7)
France	4946 (911, 8011)	4959 (1187, 7939)	9905 (2054, 16027)	39.0 (7.4, 62.3)	41.9 (10.2, 64.6)	40.3 (8.6, 63.4)	5	8 (1, 12)	5 (1, 8)	6 (1, 10)

Germany	6388 (1034, 10523)	5917 (1313, 9413)	12305 (2324, 19763)	38.7 (6.5, 61.8)	41.2 (9.3, 64.1)	39.8 (7.7, 62.9)	8	7 (1, 12)	5 (1, 8)	6 (1, 9)
Greece	786 (168, 1273)	699 (184, 1107)	1485 (348, 2396)	37.0 (8.1, 59.2)	40.1 (11.0, 62.0)	38.4 (9.3, 60.4)	18	7 (1, 11)	4 (1, 7)	5 (1, 9)
Hungary	1123 (274, 1822)	927 (294, 1438)	2049 (575, 3269)	37.6 (9.2, 59.5)	41.7 (13.1, 63.0)	39.3 (10.8, 61.0)	12	14 (3, 23)	7 (2, 12)	10 (3, 16)
Iceland	14 (3, 22)	14 (3, 22)	27 (6, 44)	38.1 (7.1, 61.3)	40.6 (9.4, 63.1)	39.3 (8.2, 62.3)	12	5 (1, 8)	4 (1, 6)	4 (1, 7)
Ireland	263 (50, 445)	186 (45, 304)	449 (95, 741)	37.0 (6.7, 59.8)	39.4 (8.7, 61.6)	37.9 (7.5, 60.7)	20	7 (1, 12)	4 (1, 7)	5 (1, 9)
Israel	324 (82, 533)	332 (96, 528)	656 (177, 1058)	35.8 (8.1, 57.0)	38.9 (10.9, 60.3)	37.2 (9.4, 58.6)	22	6 (1, 9)	4 (1, 7)	5 (1, 8)
Italy	4878 (993, 7782)	4403 (1147, 6878)	9281 (2127, 14597)	38.2 (7.9, 61.4)	41.4 (10.9, 63.3)	39.6 (9.1, 62.2)	10	7 (1, 11)	5 (1, 7)	6 (1, 9)
Japan	12726 (3311, 20188)	13356 (4903, 20812)	26082 (8321, 40865)	37.0 (9.2, 58.3)	41.2 (14.1, 61.4)	38.9 (11.3, 59.7)	14	7 (2, 12)	5 (2, 8)	6 (2, 10)
Latvia	135 (19, 225)	156 (31, 254)	291 (50, 480)	39.9 (5.8, 63.5)	42.7 (8.4, 65.9)	41.3 (7.1, 64.8)	3	9 (1, 16)	6 (1, 9)	7 (1, 11)
Lithuania	209 (30, 345)	226 (48, 359)	434 (78, 700)	40.4 (6.1, 64.5)	43.2 (9.0, 66.8)	41.8 (7.5, 65.7)	2	10 (1, 16)	6 (1, 9)	7 (1, 11)
Luxembourg	34 (6, 55)	32 (7, 51)	67 (12, 107)	38.8 (6.6, 62.1)	41.2 (9.1, 63.9)	39.9 (7.7, 63.1)	7	7 (1, 11)	5 (1, 8)	6 (1, 9)
Mexico	1973 (597, 3279)	1913 (696, 2917)	3886 (1303, 6170)	33.8 (10.5, 53.6)	37.6 (14.2, 56.3)	35.6 (12.0, 54.9)	27	3 (1, 6)	3 (1, 4)	3 (1, 5)
Netherlands	1541 (276, 2477	1327 (310, 2133)	2868 (577, 4623)	37.4 (7.2, 59.8)	40.0 (9.4, 62.5)	38.6 (8.2, 61.1)	16	9 (2, 15)	6 (1, 10)	8 (2, 12)
New Zealand	340 (87, 547)	322 (102, 498)	662 (192, 1028)	39.5 (10.0, 61.7)	43.5 (13.9, 65.4)	41.3 (11.7, 63.5)	3	9 (2, 14)	7 (2, 10)	8 (2, 12)
Norway	395 (62, 630)	425 (91, 662)	820 (149, 1291)	40.0 (6.3, 63.3)	42.6 (9, 65.7)	41.3 (7.6, 64.5)	3	8 (1, 13)	7 (1, 10)	7 (1, 12)
Poland	3605 (883, 5866)	3196 (1008, 4896)	6801 (1903, 10813)	35.8 (8.9, 57.2)	39.3 (12.1, 60.1)	37.4 (10.4, 58.7)	21	12 (3, 20)	7 (2, 11)	9 (3, 14)

Portugal	969 (223, 1549)	814 (251, 1283)	1783 (467, 2818)	36.6 (8.9, 58.3)	40.2 (12.5, 61.7)	38.2 (10.5, 59.8)	19	9 (2, 14)	5 (1, 8)	7 (2, 10)
Republic of Korea	2277 (619, 3808	2057 (745, 3436)	4334 (1406, 7197)	34.8 (9.5, 55.3)	39.8 (14.9, 59.2)	37.1 (11.9, 57.1)	23	6 (2, 10)	4 (1, 6)	5 (2, 8)
Slovakia	186 (47, 307)	194 (65, 302)	954 (249, 1530)	38.2 (9.7, 60.2)	42.3 (13.8, 63.6)	39.9 (11.1, 61.7)	7	14 (3, 23)	7 (2, 12)	10 (3, 16)
Slovenia	536 (122, 880)	419 (119, 688)	308 (58, 512)	38.0 (6.8, 60.9)	40.8 (9.5, 63.5)	39.2 (7.9, 62.0)	13	9 (2, 15)	4 (1, 7)	6 (1, 11)
Spain	178 (29, 299)	130 (29, 214)	7332 (1805, 11511)	38.9 (8.3, 61.6)	42.6 (12.2, 64.6)	40.3 (9.8, 62.9)	5	9 (2, 14)	5 (1, 8)	7 (2, 10)
Sweden	4125 (893, 6501)	3207 (917, 5019)	1400 (288, 2264)	39.1 (6.4, 62.5)	41.4 (8.8, 64.6)	40.2 (7.5, 63.6)	6	6 (1, 10)	5 (1, 9)	6 (1, 9)
Switzerland	675 (123, 1117)	724 (163, 1167)	821 (186, 1329)	38.0 (7.4, 60.9)	40.8 (9.9, 63.3)	39.3 (8.5, 62.0)	12	5 (1, 8)	3 (1, 5)	4 (1, 7)
Türkiye	434 (88, 706)	388 (97, 618)	3957 (1236, 6428)	32.5 (9.3, 51.7)	36.2 (12.8, 54.8)	34.1 (10.8, 53.1)	28	5 (2, 9)	4 (1, 6)	4 (1, 7)
United Kingdom	2218 (636, 3772)	1739 (600, 2772)	8801 (1806, 14038)	38.4 (6.7, 61.1)	41.4 (9.6, 63.8)	39.8 (8.1, 62.4)	8	7 (1, 12)	5 (1, 9)	6 (1, 10)
USA	4442 (790, 7129)	4358 (1038, 6819)	30257 (6050, 47938)	39.1 (6.4, 62.6)	43.2 (9.0, 66.8)	40.3 (7.9, 63.5)	5	6 (1, 10)	4 (1, 7)	5 (1, 8)

CRC = colorectal cancer; UI = uncertainty interval; ASDR = age-standardised death rate; OECD = Organisation for Economic Co-operation and Development. * OECD countries were ranked highest to lowest based on the age-standardised percentage of diet-related CRC deaths

Supplementary Table S3: Diet-related number, percentage, and ASR of DALYs in 2021 among OECD countries

Countries	Number (95% UI)			Age-standardized percentage (95% UI)				ASR DALYs rate (95% UI)		
	Male	Female	Both	Male	Female	Both	Rank*	Male	Female	Both
OECD	1693361 (347788, 2679269)	1395142 (377916, 2155394)	3088502 (722609, 4844604)	37.8 (7.7, 60.1)	40.9 (11.1, 62.7)	39.1 (9.1, 62.3)		155 (32, 246)	106 (28, 163)	129 (30, 202)
Australia	29313 (5708, 47205)	24247 (6190, 38891)	53560 (11898, 85652)	38.4 (7.3, 61.3)	41.4 (10.2, 63.9)	39.7 (8.6, 62.6)	10	146 (28, 234)	107 (27, 170)	125 (28, 201)
Austria	9660 (1802, 15560)	7438 (1731, 11781)	17098 (3535, 27380)	37.4 (7.1, 60.0)	40.2 (9.7, 62.5)	38.6 (8.1, 61	15	123 (23, 199)	76 (17, 120)	98 (20, 157)
Belgium	14661 (2595, 23823)	12433 (2787, 19621)	27094 (5386, 43031)	38.7 (7, 61.8)	41.4 (9.6, 64)	39.9 (8.1, 62.7)	9	144 (25, 233)	100 (22, 159)	120 (23, 192)
Canada	45541 (9077, 74084)	37973 (10025, 60018)	83514 (18914, 134678)	37.6 (7.8, 60.4)	40.4 (11.3, 62.4)	38.8 (9.3, 61.1)	14	146 (29, 237)	103 (27, 162)	123 (28, 199)
Chile	15912 (3950, 25441)	15935 (4981, 24251)	31846 (8928, 49564)	41.2 (10.7, 64)	44.8 (14.6, 66.3)	42.9 (12.5, 65.2)	1	138 (34, 221)	115 (36, 174)	126 (35, 195)
Colombia	23393 (6644, 38807)	28720 (10220, 44932)	52113 (17201, 82519)	34.5 (10.8, 54)	38.6 (14.7, 57.7)	36.6 (12.8, 56)	22	93 (27, 155)	96 (34, 150)	95 (31, 150)
Costa Rica	3631 (1002, 5947)	3617 (1239, 5643)	7248 (2230, 11553)	33.6 (9.7, 53.2)	37.7 (13.5, 56.7)	35.5 (11.4, 55)	23	143 (40, 234)	122 (42, 190)	132 (41, 210)
Czechia	20351 (4345, 34222)	13652 (3696, 21679)	34002 (8091, 56056)	35.6 (7.4, 57.7)	38.7 (10.5, 60.2)	36.8 (8.6, 58.7)	21	219 (47, 369)	119 (31, 189)	164 (38, 271)
Denmark	9511 (1689, 15392)	8427 (1902, 13353)	17938 (3532, 28801)	38.5 (6.9, 61.8)	41.1 (9.3, 63.9)	39.7 (8, 62.8)	10	174 (31, 282)	133 (30, 210)	153 (30, 245)
Estonia	2149 (281, 3564)	1926 (341, 3122)	4075 (623, 6651)	39.4 (5.5, 63.2)	41.8 (7.9, 65.2)	40.5 (6.5, 64.2)	6	215 (28, 356)	118 (20, 190)	155 (23, 252)
Finland	6077 (1110, 9981)	5296 (1195, 8451)	11372 (2282, 18457)	37.4 (7.0, 60.4)	39.8 (9.3, 62)	38.5 (7.9, 61.1)	16	111 (20, 182)	79 (18, 127)	94 (19,152)
France	95649 (17023, 154096	81125 (18642, 129346	176774 (35873, 283700	39.3 (7.2, 62.7)	42.0 (9.9, 64.8)	40.5 (8.3, 63.7)	5	163 (28, 262)	105 (24, 167)	131 (26, 210)

Germany	130215<br>(20530, 213151)	103766<br>(21926, 166064)	233980<br>(42468, 377323)	38.9<br>(6.4, 62.4)	41.4<br>(9, 64.4)	39.9<br>(7.4, 63.3)	9	158<br>(25, 257)	104<br>(21, 165)	129<br>(23, 209)
Greece	14617<br>(2968, 23721	11702<br>(2983, 18534)	26319<br>(5988, 42194)	37.4<br>(7.8, 59.5)	40.3<br>(10.6, 62.4)	38.6<br>(8.9, 60.7)	15	141<br>(28, 228)	93<br>(23, 146)	115<br>(26, 183)
Hungary	25899<br>(6108, 42023)	18592<br>(5771, 28904)	44491<br>(12098, 70563)	37.7<br>(9.0, 59.7)	41.7<br>(12.9, 63.2)	39.3<br>(10.5, 61.1)	12	332<br>(78, 539)	174<br>(53, 270)	242<br>(65, 384)
Iceland	285<br>(51, 464)	254<br>(58, 410)	539<br>(109, 874)	38.4<br>(6.9, 61.7)	40.8<br>(9.2, 63.7)	39.5<br>(8, 62.7)	11	105<br>(19, 171)	85<br>(19, 137)	95<br>(19, 154)
Ireland	5536<br>(989, 9355)	3625<br>(866, 5832)	9161<br>(1863, 15075)	37.3<br>(6.5, 60.3)	39.6<br>(8.6, 62.1	38.2<br>(7.3, 61.1	18	150<br>(27, 254)	88<br>(21, 142)	118<br>(24, 194)
Israel	6471<br>(1588, 10766)	5846<br>(1715, 9406)	12317<br>(3310, 20045)	36.0<br>(7.9, 57.5)	39.0<br>(10.6, 60.5)	37.3<br>(9.1, 58.9)	20	116<br>(28, 193)	88<br>(25, 141)	101<br>(27, 165)
Italy	93866<br>(18847, 149033)	75964<br>(19395, 118720)	169830<br>(38242, 266960)	38.4<br>(7.6, 61.6)	41.5<br>(10.5, 63.6)	39.7<br>(8.8, 62.5)	10	152<br>(30, 241)	99<br>(25, 154)	123<br>(27, 192)
Japan	247222<br>(61047, 389972)	208548<br>(71401, 320546)	455770<br>(134725, 709276)	37.6<br>(9.1, 59.3)	41.8<br>(13.7, 62.4)	39.3<br>(10.9, 60.6)	12	172<br>(42, 271)	113<br>(37, 171)	141<br>(39, 217)
Latvia	2952<br>(393, 4919)	2943<br>(567, 4814)	5895<br>(961, 9689)	40.2<br>(5.6, 64)	42.8<br>(8.1, 66.1)	41.4<br>(6.8, 65.1)	4	209<br>(28, 348)	126<br>(24, 207)	158<br>(25, 260)
Lithuania	4498<br>(624, 7331)	4200<br>(839, 6653)	8699<br>(1464, 13964)	40.6<br>(5.8, 64.8	43.4<br>(8.5, 67.1	41.9<br>(7.1, 66)	2	216<br>(30, 353)	124<br>(24, 197)	159<br>(26, 255))
Luxembo urg	722<br>(111, 1152)	559<br>(111, 887)	1281<br>(219, 2030)	39.2<br>(6.4, 62.8)	41.5<br>(8.7, 64.6)	40.2<br>(7.3, 63.7)	7	147<br>(23, 235)	96<br>(19, 153)	121<br>(20, 191)
Mexico	54100<br>(15876, 90476)	49381<br>(17677, 75896)	103481<br>(33582, 165002)	33.6<br>(10.1, 53.6)	37.5<br>(13.8, 56.2)	35.4<br>(11.6, 54.8)	24	88<br>(26, 147)	71<br>(25, 109)	79<br>(26, 126)
Netherlan ds	32723<br>(5677, 52560)	25980<br>(5886, 41807)	58703<br>(11593, 94146)	37.7<br>(7.0, 60.3)	40.2<br>(9.2, 62.9)	38.8<br>(7.9, 61.5)	14	204<br>(35, 327)	145<br>(33, 233)	172<br>(34, 277)
New Zealand	7078<br>(1772, 11197)	6170<br>(1947, 9404)	13248<br>(3730, 20797)	39.8<br>(9.7, 62.2)	43.7<br>(13.6, 65.8	41.5<br>(11.4, 63.8)	3	184<br>(46, 291)	142<br>(45, 216)	162<br>(45, 255)
Norway	7931<br>(1203, 12567)	7241<br>(1441, 11157)	15173<br>(2627, 23707)	40.4<br>(6.1, 63.9)	42.8<br>(8.6, 66.2)	41.5<br>(7.2, 64.9)	3	172<br>(26, 272)	135<br>(26, 208)	153<br>(26, 239)
Poland	78173<br>(18799, 126880	59124<br>(18436, 89713)	137297<br>(37548, 218480)	36.0<br>(8.7, 57.5)	39.4<br>(11.8, 60.4)	37.4<br>(10.0, 58.8)	19	262<br>(63, 425)	145<br>(45, 220)	196<br>(53, 311)
Portugal	19344<br>(4271, 31165)	14159<br>(4103, 22271)	33503<br>(8236, 53029)	36.9<br>(8.6, 58.7)	40.4<br>(12.0, 62.0)	38.3<br>(9.9, 60.0)	17	193<br>(42, 312)	109<br>(30, 170)	146<br>(35, 232)
Republic of Korea	51785<br>(13427, 86562)	38643<br>(14368, 64805)	90428<br>(27166, 151428	34.9<br>(9.2, 55.6)	39.5<br>(14.2, 59.2)	36.8<br>(11.1, 57)	21	124<br>(33, 209)	77<br>(29, 130)	99<br>(30, 165)
Slovakia	12501	8686	21188	38.3	42.4	40.0	8	305	164	225

	(2801, 20403)	(2401, 14309)	(5375, 34094)	(9.4, 60.6)	(13.4, 63.8)	(10.7, 61.9)		(69, 497)	(45, 271)	(57, 362)
Slovenia	3556	2196	5752	38.2	40.9	39.2	13	186	89	133
	(578, 5996)	(477, 3651)	(1052, 9567)	(6.7, 61.3)	(9.1, 63.6)	(7.5, 62.2)		(30, 314)	(19, 148)	(24, 221)
Spain	81347	56319	137666	39.0	42.6	40.4	6	193	107	146
	(17294, 127650)	(16013, 87562)	(32843, 216061)	(8.0, 61.9)	(11.8, 64.8)	(9.4, 63.1)		(41, 303)	(30, 166)	(34, 229)
Sweden	12652	12191	24843	39.3	41.7	40.4	6	129	109	119
	(2248, 21006)	(2670, 19524)	(5007, 40676)	(6.2, 63)	(8.7, 64.8)	(7.3, 63.9)		(23, 213)	(23, 176)	(23, 193)
Switzerland	8470	6858	15328	38.3	41.0	39.5	11	104	72	87
	(1664, 13636)	(1666, 10874)	(3353, 24440)	(7.2, 61.3)	(9.6, 63.6)	(8.2, 62.3)		(20, 166)	(17, 113)	(19, 137)
Türkiye	55754	40147	95901	32.4	35.9	33.8	25	124	81	101
	(15891, 94265)	(13552, 63425)	(29451, 157132)	(9.0, 51.6)	(12.4, 54.7)	(10.4, 52.8)		(36, 210)	(27, 128)	(31, 166)
United Kingdom	88291	77658	165949	38.7	41.6	40.0	8	154	116	134
	(15153, 141163)	(17569, 121178)	(32736, 263323)	(6.5, 61.6)	(9.4, 64.3)	(7.7, 62.8)		(26, 246)	(26, 182)	(26, 213)
USA	371525	313601	685126	39.4	41.9	40.5	5	149	108	128
	(59838, 591495)	(70110, 485798)	(130008, 1077155)	(6.1, 63)	(9.2, 64.8)	(7.5, 63.9)		(24, 238)	(24, 167)	(24, 201)

CRC = colorectal cancer; UI = uncertainty interval; ASR = age-standardised rate; DALYs= disability-adjusted life years; OECD = Organisation for Economic Co-operation and Development. * OECD countries were ranked highest to lowest based on the age-standardised percentage of diet-related CRC DALYs

Supplementary Table S4: Burden (deaths and DALYs) of CRC attributable to specific dietary risk factors in OECD countries

Dietary risks	Deaths (95% UI)			DALYs (95% UI)		
	Number	Crude rate per 100000	Crude proportion (%)	Number	Crude rate per	Crude proportion (%)
Diet low in whole grains	71355 (29764, 108138)	5.2 (2.2,7.9)	18.0 (7.6, 26.8)	1420579 (596153, 2116475)	103.5 (43.4,154.2)	18.0 (7.5, 26.8)
Diet low in fiber	4951 (2264, 7655)	0.4 (0.2,0.6)	1.3 (0.6, 1.9)	89876 (40458, 139802)	6.5 (2.9,10.2)	1.1 (0.5, 1.8)
Diet high in processed meat	35375 (-8577, 71939)	2.6 (-0.6,5.2)	8.9 (-2.2, 18.3)	739804 (-182962, 1495670)	53.9 (-13.3,109)	9.4 (-2.4, 19.1)
Diet low in milk	46648 (12285, 78664)	3.4 (0.9,5.7)	11.8 (3.1, 19.4)	894550 (235712, 1499071)	65.2 (17.2,109.2)	11.3 (3, 18.7)
Diet high in red meat	61546 (-23, 123382)	4.5 (0,9.0)	15.5 (0, 31.1)	1240390 (-500, 2463818)	90.4 (0,179.5)	15.7 (0, 31.4)
Diet low in calcium	17966 (12435, 23947)	1.3 (0.9,1.7)	4.5 (3.2, 5.9)	326230 (229576, 430943)	23.8 (16.7,31.4)	4.1 (3, 5.4)

CRC = colorectal cancer; OECD = Organisation for Economic Co-operation and Development; DALYs = disability-adjusted life years

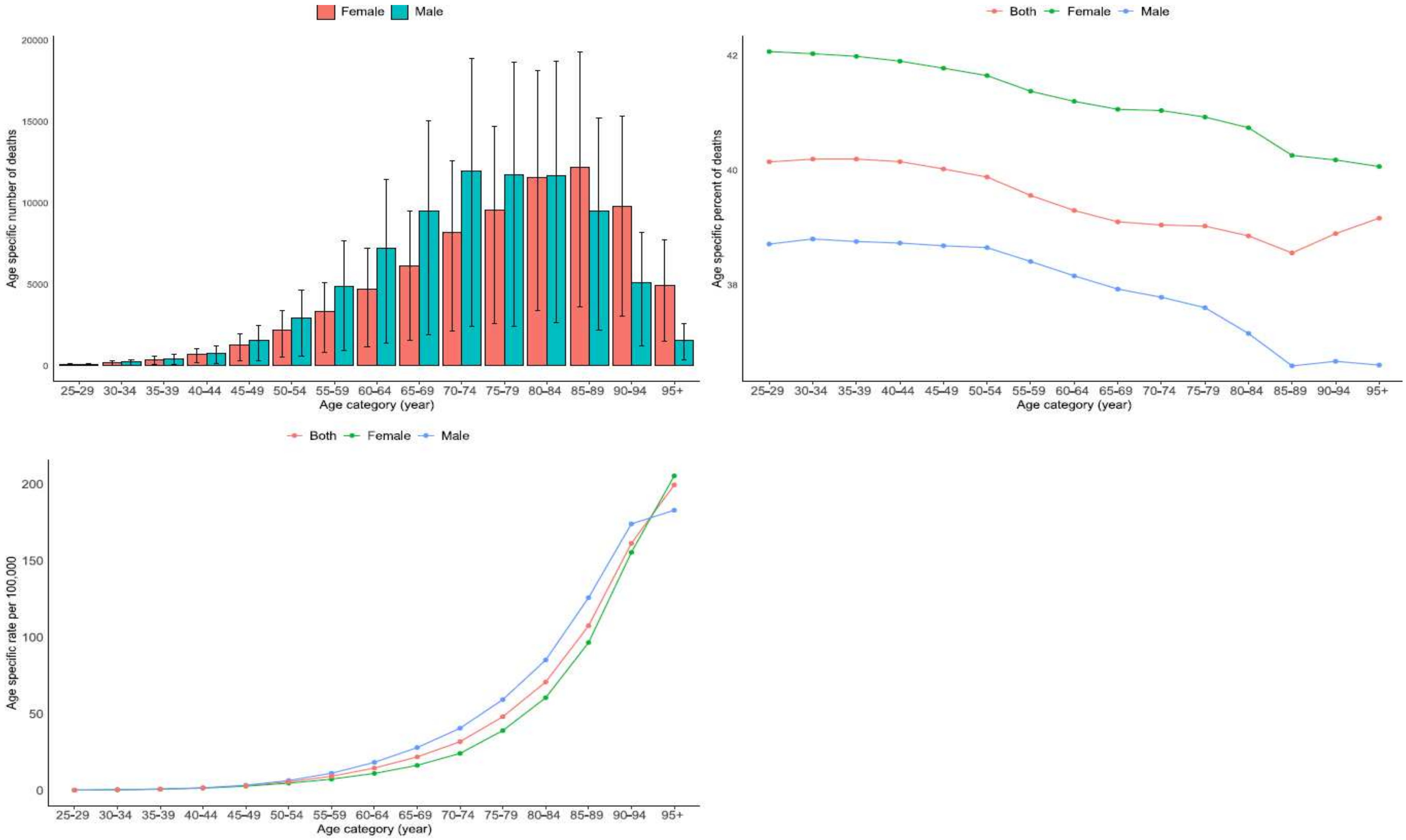

Figure S1: Diet-related CRC deaths across age groups in 2021

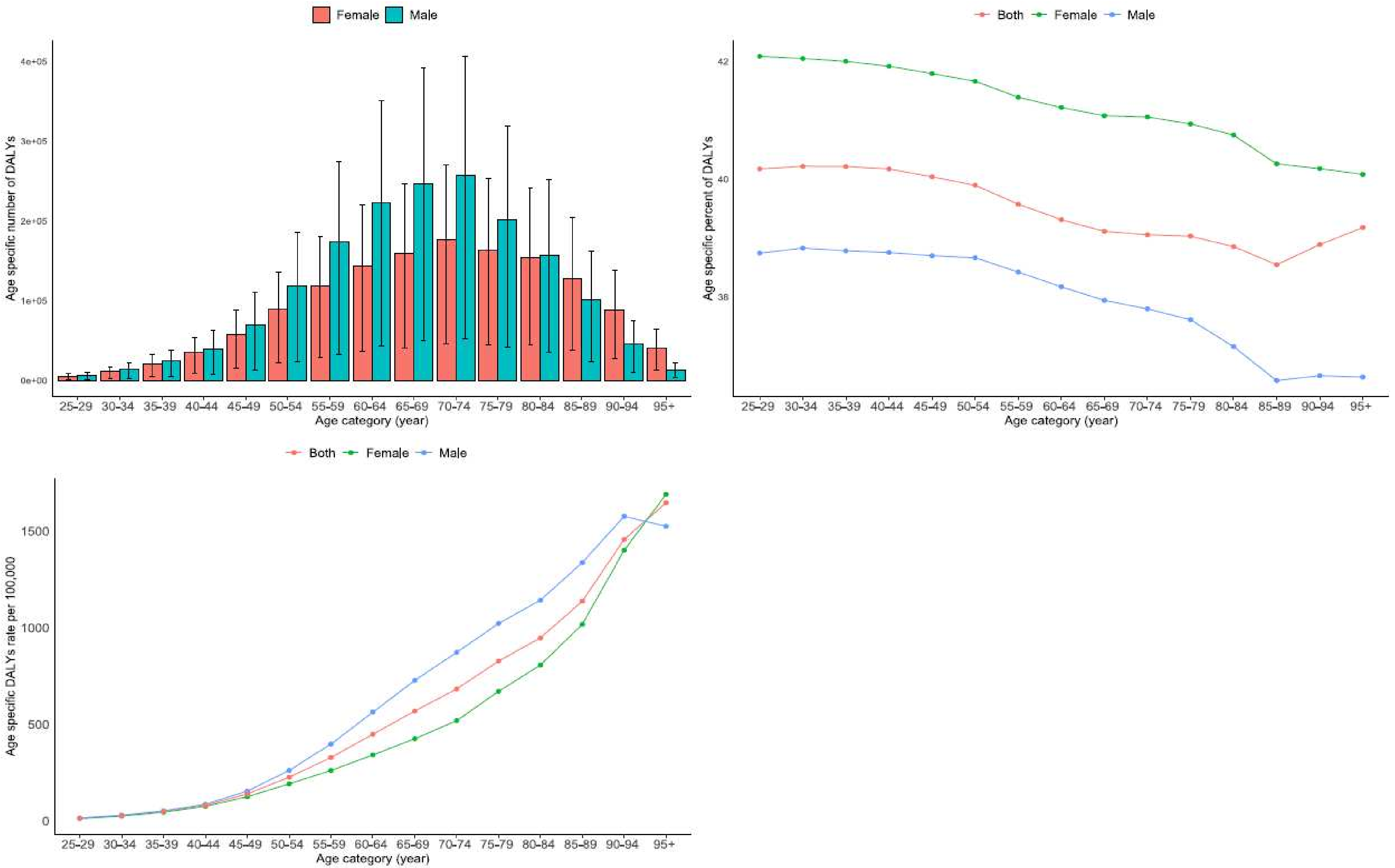

Figure S2: Diet-related CRC DALYs by age groups in 2021

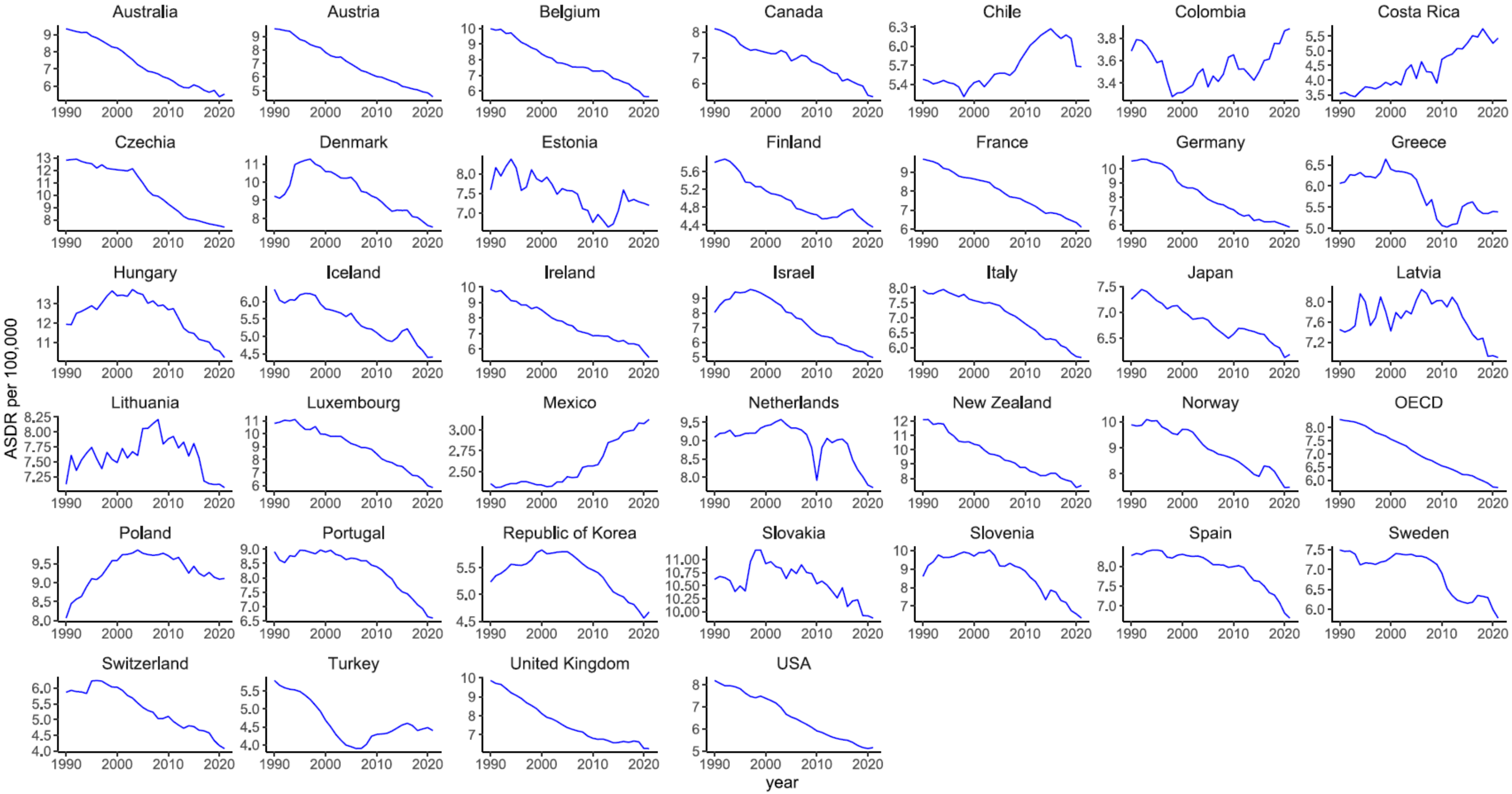

Figure S3: Trend of diet-related ASDR from 1990 to 2021

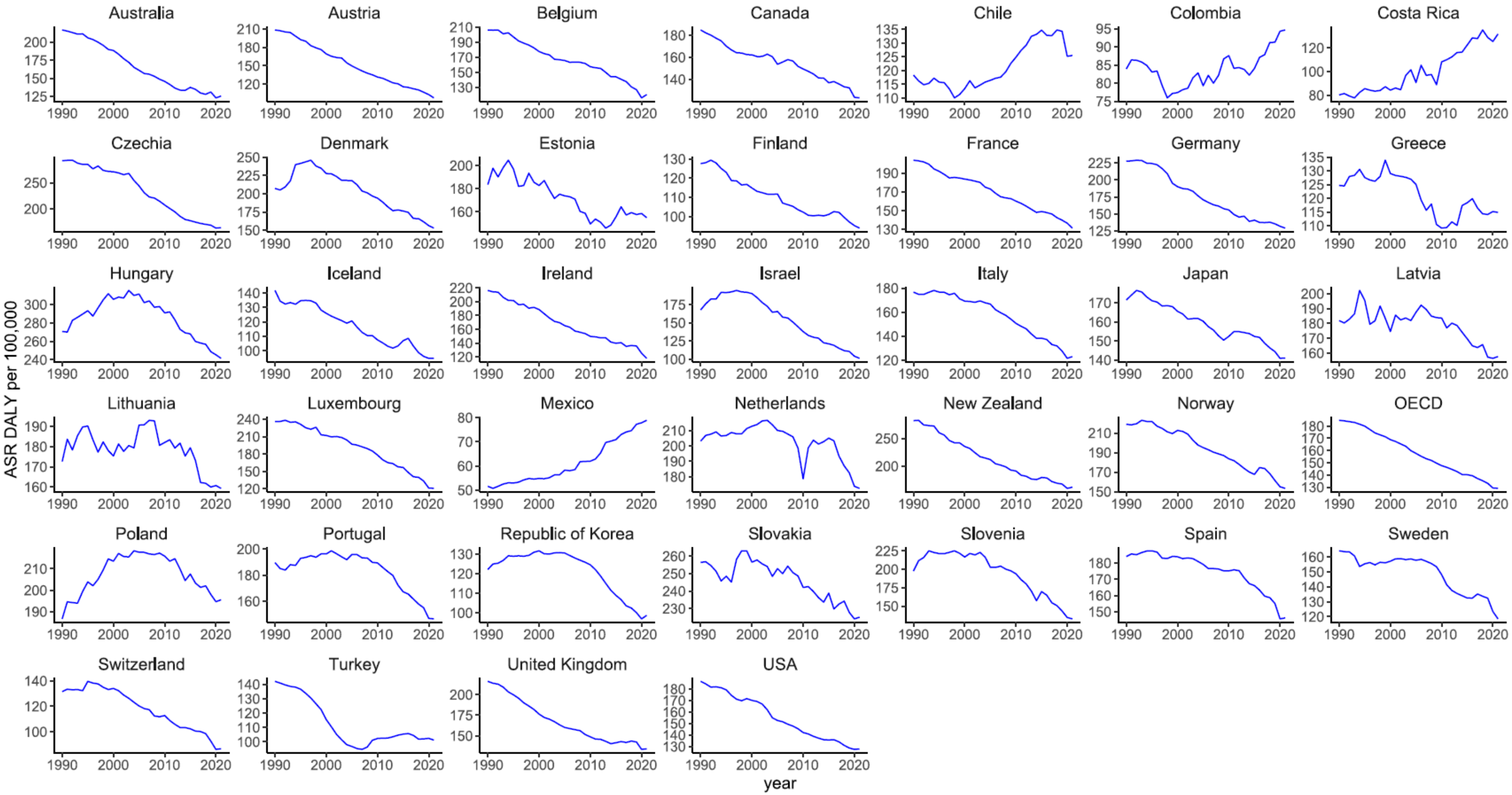

Figure S4: Trend of diet-related ASR rate of DALYs from 1990 to 2021

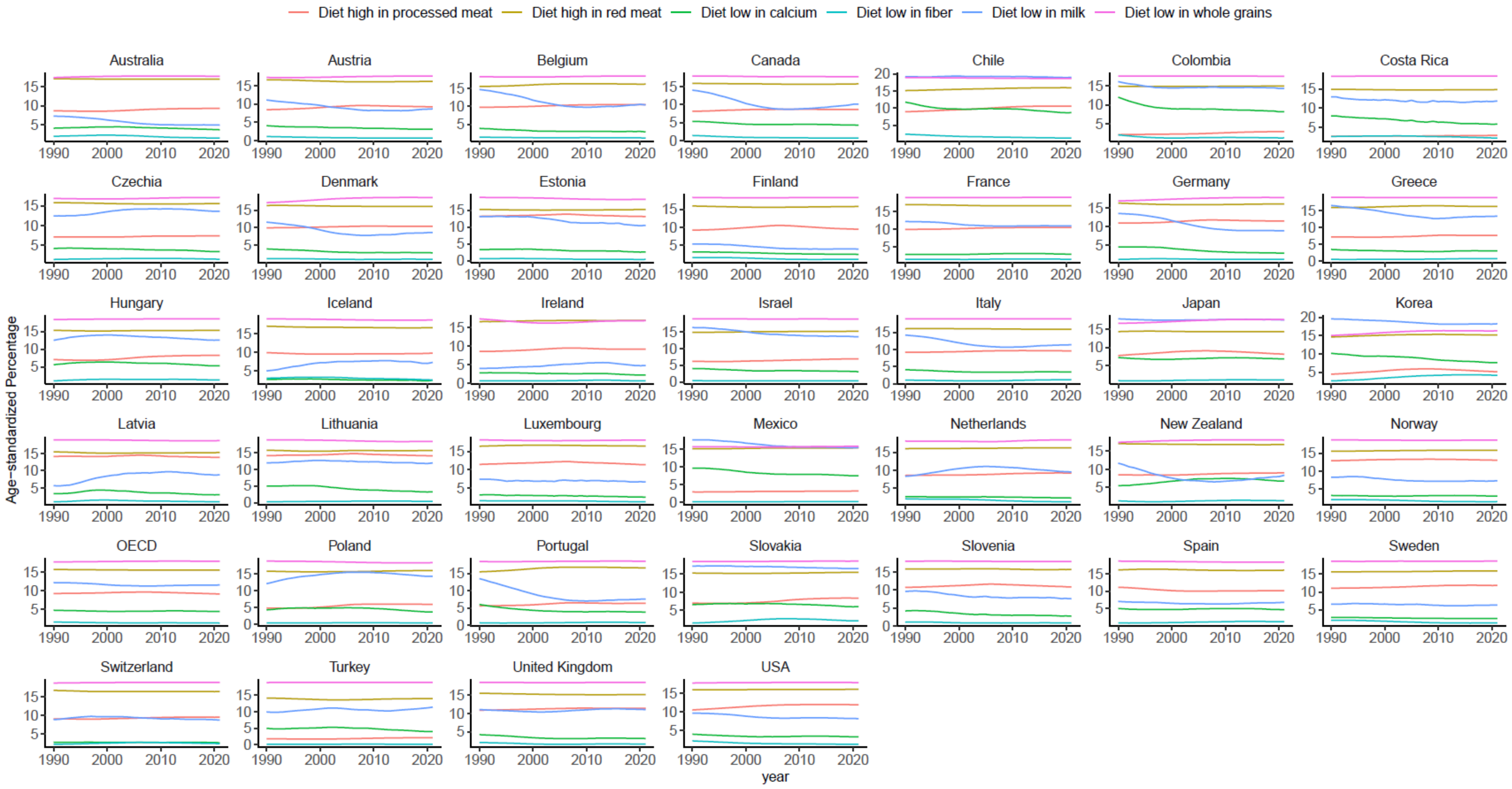

Figure S5: Trends of specific diet-related CRC deaths among OECD countries from 1990 to 2021

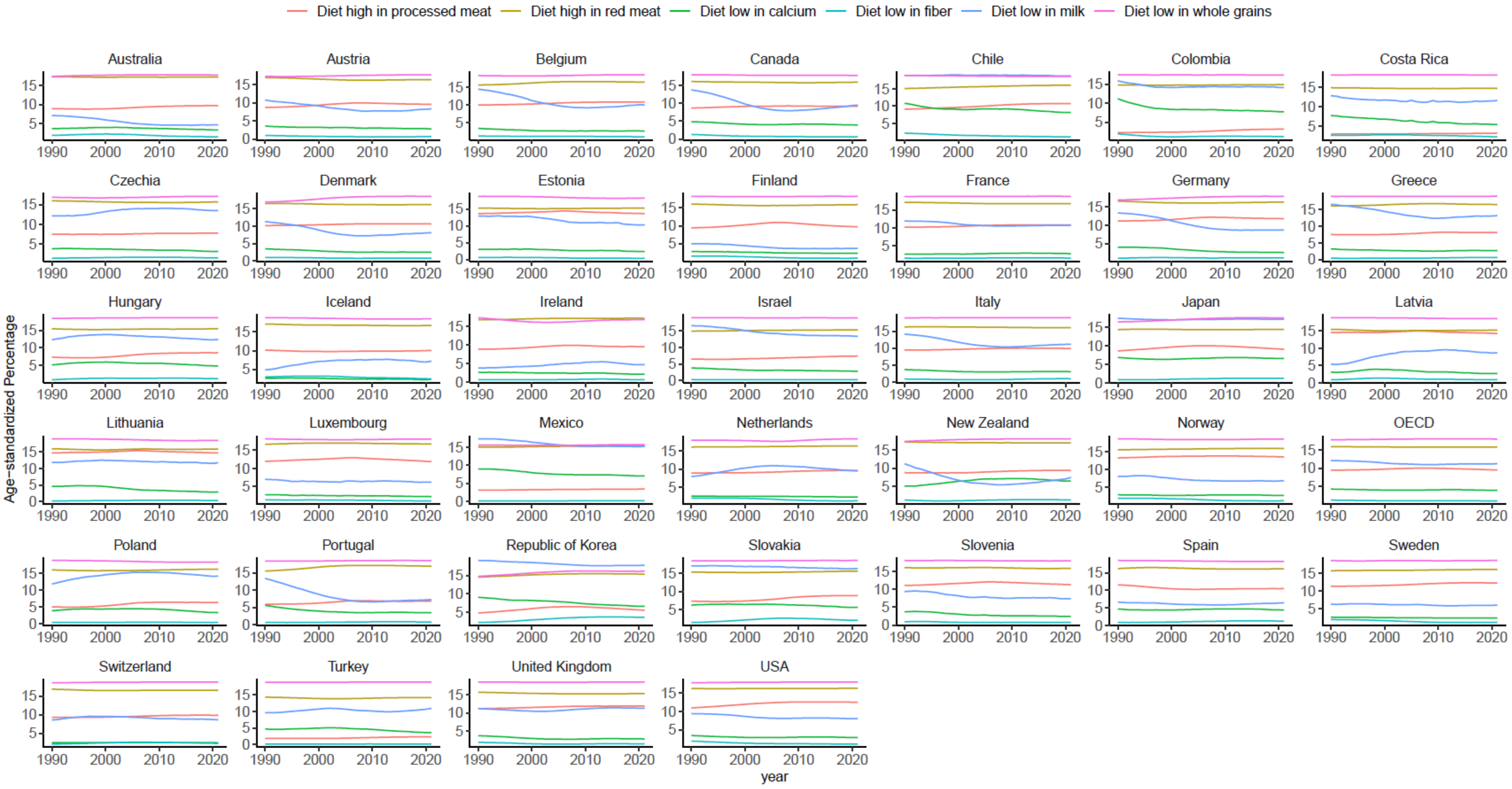

Figure S6: Trends of specific diet-related CRC DALYs among OECD countries from 1990 to 2021
